# UHRF1 is a sensor for DNA interstrand crosslinks

**DOI:** 10.18632/oncotarget.6647

**Published:** 2015-12-17

**Authors:** Chih-Chao Liang, Martin A. Cohn

**Affiliations:** Department of Biochemistry, University of Oxford, Oxford, United Kingdom

**Keywords:** fanconi anemia, ICL repair, UHRF1, FANCD2, ubiquitin

The human genome is guarded against mutations by several DNA repair pathways. Common for all pathways, is the initiating event of detecting the DNA damage. This initial event is followed by the coordinated recruitment of various other DNA repair proteins needed for the repair. For instance, the MSH2/MSH6 complex detects DNA mismatches, while the MRN or KU70/KU80 complexes detect double strand breaks. We have recently identified the UHRF1 protein as a sensor for DNA interstrand crosslinks (ICLs) [1].

DNA interstrand crosslinks (ICLs) are extremely toxic to cells. In human, the Fanconi Anemia (FA) DNA repair pathway repairs such DNA damage [2]. Disruption of the FA pathway in human causes the FA disease, where patients are characterized by developmental abnormalities, bone marrow failure, and a high incidence of cancer. To date, 19 FA genes have been identified. In addition, a number of other gene products cooperate in the FA pathway. In addition to the critical step of detecting the damage, repair of an ICL entails incision of the ICL by nucleases, translesion synthesis, nucleotide excision repair and homologous recombination.

The ICL repair reaction has been studied extensively in various model systems. An ICL can be an obstacle to DNA replication, and the convergence of two replication forks from opposite sides of the ICL can trigger the repair reaction as shown in the Xenopus cell free system [3]. At the same time, traverse through an ICL by the replication machinery has been reported in mammalian cells, leaving an unrepaired ICL for later repair [4]. When the MRN complex is recruited to a DNA double strand break, histone H2AX is phosphorylated by ATM, which allows the recruitment of MDC1, RNF8 and RNF168. RNF8 mediates ubiquitination of histone H1 and RNF168 mediates ubiquitination of histone H2A, creating a landing platform to subsequently recruit downstream factors [5]. Unlike the double strand break repair pathway, an actual ICL sensor protein has remained unknown.

The identification of UHRF1 opens the possibility that the initial stages of ICL repair might be conceptually similar to the repair of other types of DNA damage. UHRF1 interacts with ICLs both *in vitro* and *in vivo*. Using live-cell imaging, we established that UHRF1 is recruited to ICLs within seconds after their appearance in the cell, kinetics similar to that of early stage repair proteins in other DNA repair pathways. The initiating event of UHRF1 recruitment facilitates the subsequent recruitment of the key FA protein, FANCD2. It is currently unclear how UHRF1 mediates this event. It is possible that a direct interaction between the two proteins exists. It has also been suggested that UHRF1 interacts with nucleases, and this might be important for its role early in the ICL repair process [6].

The discovery of UHRF1 as an ICL sensor prompts several questions. For instance, we do not know the nature of the interaction with an ICL. Structural studies will be required to uncover this. Also, it will be interesting to clarify whether the function of UHRF1 is required for ICL repair in a replication-dependent or -independent manner. Association of UHRF1 with an ICL in the G1-phase of the cell cycle could potentially mark the crosslink for later repair when the cell enters S-phase, and might prevent other DNA repair pathways from initiating repair prior to the FA pathway.

DNA repair pathways are often deregulated in cancer. Therefore, increased understanding of these pathways could accelerate the development of new therapeutic approaches. UHRF1 is deregulated in a number of cancers, suggesting that it might be a potential therapeutic target [7]. Also, ICL-forming drugs, such as cisplatin and mitomycin C, are currently used effectively in the treatment of various types of cancer. However, occurrence of resistance is a major problem. It is possible that the developed resistance is caused by increased cellular ability to repair ICLs. Therefore, targeting ICL repair pathways directly could potentially sensitize cancer cells to existing therapeutic drugs. Future research will hopefully address these questions.
